# Unity in diversity, a systems approach to regulating plant cell physiology by 2-oxoglutarate-dependent dioxygenases

**DOI:** 10.3389/fpls.2015.00098

**Published:** 2015-03-11

**Authors:** Siddhartha Kundu

**Affiliations:** School of Computational and Integrative Sciences, Jawaharlal Nehru UniversityNew Delhi, India

**Keywords:** dioxygenases, 2-oxoglutarate, facial triad, systems biology, hidden Markov model, generalized linear model

## Abstract

Could a disjoint group of enzymes synchronize their activities and execute a complex multi-step, measurable, and reproducible response? Here, I surmise that the alpha-ketoglutarate dependent superfamily of non-haem iron (II) dioxygenases could influence cell physiology as a cohesive unit, and that the broad spectra of substrates transformed is an absolute necessity to this portrayal. This eclectic group comprises members from all major taxa, and participates in pesticide breakdown, hypoxia signaling, and osmotic stress neutralization. The oxidative decarboxylation of 2-oxoglutarate to succinate is coupled with a concomitant substrate hydroxylation and, in most cases, is followed by an additional specialized conversion. The domain profile of a protein sequence was used as an index of miscellaneous reaction chemistry and interpreted alongside existent kinetic data in a linear model of integrated function. Statistical parameters were inferred by the creation of a novel, empirically motivated flat-file database of over 3800 sequences (DB2OG) with putative 2-oxoglutarate dependent activity. The collated information was categorized on the basis of existing annotation schema. The data suggests that 2OG-dependent enzymes incorporate several desirable features of a systems level player. DB2OG, is free, accessible without a login to all users, and available at the following URL (http://comp-biol.theacms.in/DB2OG.html).

## Introduction

Physiology is an unmeasured outcome of a complex undefined molding of several underlying, disparate, and interdependent molecular level events. A particularly challenging problem is harmonizing these seemingly unrelated micro-steps to accomplish a macroscopic scale observable. The architecture of any such model would have to be sufficiently coarse-grained to take into account the magnitude and dimensions of the predicted response, yet be offset by a small set of regulatory parameters which could function as reaction modifiers. Finally, given the inherent complexity of biological systems and the varied approximations of existent models, a cumulative error term that summarizes inestimable and excluded data points is an obligatory element of any representation under consideration. In biochemical terms this would be analogous to an enzyme system which, as a unit could catalyze the conversion of several different substrates using a library of reaction chemistries, and yet possess similar kinetics with common co-factors, -enzymes, and/or—substrates. Miscellaneous contributors such as subcellular location, feedback mechanisms, number of irreversible reactions, and presence of pathways with common metabolic intermediates would also need to be factored in. A reasonable candidate appears to be the 2OG-dependent superfamily of enzymes.

The alpha-ketoglutarate (AKG) and non-heme Fe (II) dependent dioxygenases catalyze the incorporation of both atoms of molecular dioxygen in a reaction which may be described as an oxidative decarboxylation with associated hydroxylation. In these enzymes, water and 2OG function as uni- and bi-dentate ligands. The residues His-X-Asp/Glu-Xn-His form dative linkages with iron at one face of a regular octahedron, distorting it completely. This arrangement, however, may aptly be described as combinatorial, with other dioxygenases sharing a similar conserved motif-homology (Hegg and Que, 1997). The specific and fixed inter-residue metrics of the HD/EH-motif along with arginine (Arg; R), tryptophan (Trp; W), tyrosine (Tyr; Y), asparagine (Asn; N), glutamine (Gln; Q), serine (Ser; S), threonine (Thr; T), and methionine (Met; M) complete the active site of most 2OG-dependent enzymes. The superfamily is also unique for its substrate driven specialized reaction chemistry, and encompasses chlorination, desaturation, epoxidation, aliphatic- and aromatic- ring closure, and lysine demethylation. The relaxed coordination of iron, formation of an exceptionally reactive, transient ferryl species [Fe(IV)=O], and several sequence specific features are purported to contribute to the substrate versatility of 2OG-dependent enzymes (Price et al., 2003a,b; Hausinger, 2004; Clifton et al., 2006).

The major focus of this work was to numerically derive a mathematical expression that could highlight the ability of this cluster to work in a concerted and organized manner to execute complex cellular function. A critical parameter to be considered is a measure of enzyme variability. The exemplary multiformity demonstrated by these enzymes is well-documented with biochemical, mutagenesis, and structural data available from several laboratories. Hypothetically, the distribution of AKG-catalytic domains in a particular enzyme(s) when examined against a similar spread in a larger repository of sequences might be a suitable index of this inconsonance. Since, data from characterized enzymes (*N* ≅ 227; Kundu, 2012) is limited, a larger database was sought to accomplish this. The profile HMMs from Pfam lists several candidate (>5500; PF02668_TauD) sequences and is exceptionally detailed. However, this data is based on generic models which includes sequences with unvalidated and putative function. A novel database, DB2OG, was then constructed wherein predicted catalytic domains of biochemically validated enzymes were mapped onto a set of sequences which had no supporting laboratory data. This extrapolated parameter, i.e., the simultaneous domain probability with respect to the taxa under consideration, when merged with available kinetic data could offer insights into the system being modeled.

Plant 2OG-dependent enzymatic activity is narratively charted, with genomic and proteomic data, and their variants thereof. They participate principally as hormonal regulators (gibberellic acids, jasmonic acid), general flavonoid metabolism, alkaloid biosynthesis, and maintainence of structural integrity (prolyl hydroxylases) (Kawai et al., 2014). The presence of a rigid cell wall, absence of an extracellular matrix, and the abundance of organelles constitutes a defined experimental system amenable to hypothesis testing with minimal confounders. These features were instrumental in selecting the phytocellular mileau as the system of study.

## Materials and methods

### Construction and analysis of DB2OG

The database, DB2OG, was created using a generic hidden Markov model (HMM) of alignments (Kundu, 2012) and used to query the UniprotKB database (http://uniprot.org). Briefly, members of the alpha-ketoglutarate superfamily (EC 1.14.11.x, EC 1.14.20.y) with available empirical data were randomly clustered, with at least 2–4 members of each family contributing to the model. In this work, a family is defined as enzymes with the same substrate profile. Several filters were applied to screen this initial set. The final list (*N* = 3806), comprised experimentally unvalidated full-length protein sequences. A detailed chart of the catalytic profile of these sequences was constructed using the server module of H2OGpred (http://comp-biol.theacms.in/H2OGpred.html; Kundu, 2012). This matrix of profiles (Tables T1; S1A, S1B) is used to search DB2OG for suitable matches. Analysis to ascertain the preferred intracellular location was carried out with the PSORT suite of programs (Horton et al., 2007; Yu et al., 2010; Tables T3, T4). The nomenclature of the clusters is with regards to the substrates they catalyze, and is in accordance with previous work (Kundu, 2012). Chemical structures were drawn by combing data from PubChem_Compound (http://ncbi.nlm.nih.gov/pccompound) and ChemSketch 12.00 (freeware) downloaded and installed locally. Phylogenetic trees and alignment files were generated using the STRAP suite of programs (Gille and Frommel, 2001), and all biochemical data was extracted from BRENDA (Schomburg et al., 2002).

### Implementing DB2OG as a universally accessible portal

Unlike the sequence based pairwise-scoring and threshold-exceeding search scheme, DB2OG was populated by formulating the query string as a profile-HMM. The database is organized into three sections: (a) introduction to 2-oxoglutarate dependent dioxygenases with explanations of the types of files that are accessible and general usage, (b) database search. This is subdivided into, (i) a pre-defined sequence based search, and (ii) a user-specific patterned query. The sequences were previously categorized on the basis of their taxonomic spread and their predicted cell locations. The results can be downloaded as standard fasta (^*^.fasta) files. The specialized query option is exclusively determined real-time by the user. The search criteria, which are logical functions are initially used to probe a flat-file implementation of the computed chart of profiles. The selected domains are used to retrieve the sequences. All coding was done in-house using PERL. The website was implemented with standard PERL- CGI-HTML codes. Consider the following examples for a profile-based search:

**Case a:** Identify sequences of DB2OG **with** the following SULF-, TFDA- domains, but exclude those with a PHYT-domain
(x ∈ *DB2OG*| {SULF, TFDA} ⊆ *x*; {PHYT} ⊄ *x*).
**Case b:** Identify sequences of DB2OG **with** the following SULF-, TFDA- domains
(x ∈ *DB2OG*| {SULF, TFDA} ⊆ *x*)
**Case c:** Identify sequences of DB2OG **without** a PHYT-domain
(x ∈ *DB2OG*| {PHYT} ⊄ *x*)

In these examples cases- b and -c are simple search options using logical “OR,” while case- a would constitute a complex search with a logical “AND” function.

### Mathematical models

HMMs are a sub-class of Markov models (a model where the future system state is determined by the current state and is, therefore “memoryless”), wherein, given the presence of observables, algorithms are used to compute the sequential probabilities of occurrence of hidden states (Markov chains), and are part of the online repositories Pfam and H2OGpred among several others (Sonnhammer et al., 1997; Kundu, 2012). HMMs used in this work were constructed using HMMER3.0 (http://hmmer.janelia.org) downloaded locally. The preliminary alignments as fasta (^*^.fasta) formatted files were converted to the stockholm (^*^.sto, ^*^stockholm) formats. Generalized linear models (GLMs), are mathematically, linear combinations of more than one independent variable. This abstract representation is able to hypostatize the functioning of several enzymes into a concise numerical summary which can be reverse mapped to well-established distributions such as the gaussian, poisson's, and exponential probability densities. A statistical model was formulated to incorporate known and predefined parameters (Equations 1 and 2) of enzymes that could contribute to a particular reaction. The final computations were done using the generalized linear model (GLMs) function of R-3.0.0 (S3). An algorithm outlining the steps involved in these calculations is mentioned.

## Results and discussion

### Unique features of DB2OG

#### Access and usage

**Figure 1:**
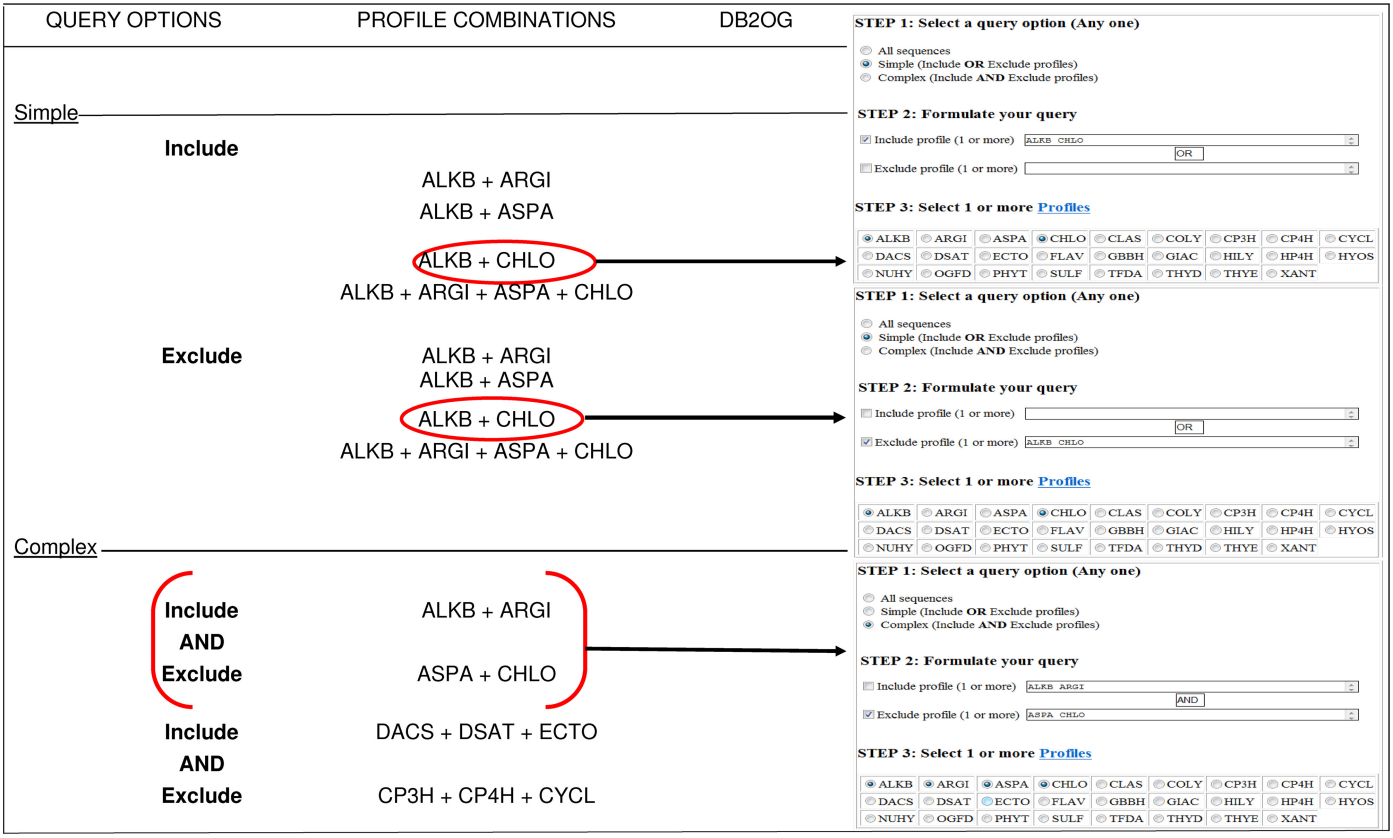
**Profile based search of sequences in DB2OG**. Users may query the database for putative 2-OG dependent sequences using one or more of the pre-defined HMM profiles (*N* = 26). A structured query of this nature (simple or complex), without an inclusion score will list all sequences with the profile of interest. Other sequences corresponding to variable subcellular locations may also be downloaded by clicking on the appropriate hyperlinks. NOTE: The browser window displaying the search results needs to be refreshed for each query.

The GUI introduces and summarizes the salient features of alpha-KG dependent enzymes, and provides general instructions of use. The user may opt to download various categories of sequences or search the database for combinations of previously computed profiles (twenty-six functional hidden Markov model profiles; Kundu, 2012). Since, most sequences of the database have more than one assigned profile, there are several examples of overlapping data. This may be minimized by usage of the logical OR/AND functions to filter the profiles (Figure 1). The matched list is presented as a simple text file, which may be downloaded or viewed online. Users are encouraged to refresh their browsers to visualize the latest search results.

#### Description and characterization of DB2OG

**Figure 2:**
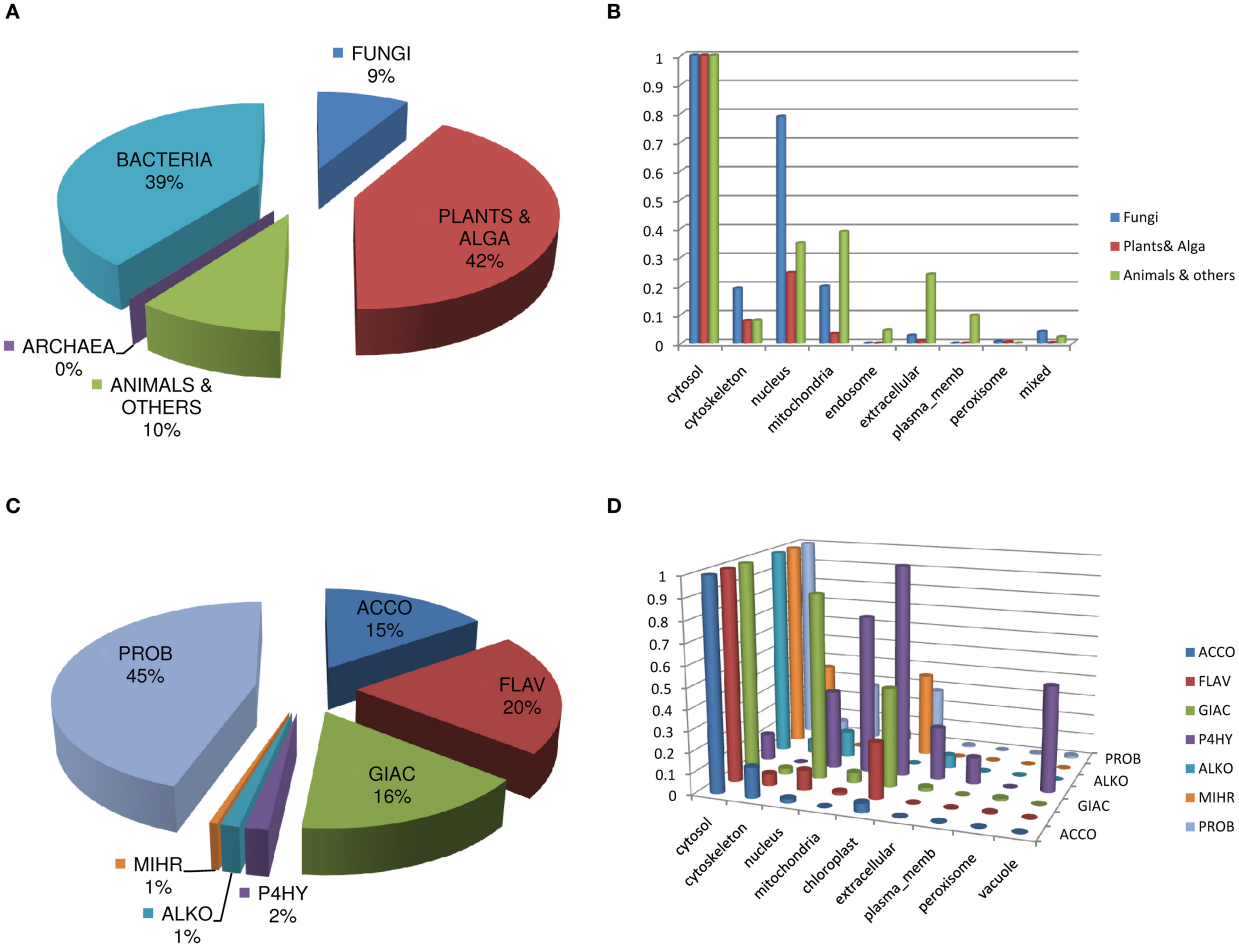
**Analysis and description of DB2OG**. **(A)** Taxonomic distribution of sequences among all known kingdoms of life, **(B)** Predicted sub-cellular location of sequences using the PSORT suite of programs. **(C)** 2-OG dependent enzyme groups in plants (*n* = 1624), with their **(D)** predicted cellular location. P4H, unlike the other groups has a compartmental preference, with appreciable numbers in the nuclear, mitochondrial, vacuolar, chloroplast, and extracellular regions of the cell. GA-oxidases have an almost comparable cytosolic and nuclear distribution. Data represent normalized values of the highest score for a particular cellular region in either plants, animal, or fungi **(C)** or 2OG-dependent enzyme clusters **(D)**. Abbreviations: ALKB, Alk-B like demethylase; ARGI, Arginine hydroxylase; ASPA, Aspartyl:Asparaginyl hydroxylase; CHLO, Chlorination; CLAS, Clavaminate synthase; COLY, Collagen lysyl dioxygenase; CP3H, Collagen prolyl 3-hydroxylase; CP4H, Collagen prolyl 4-hydroxylase; CYCL, Cyclization; DACS, Deacetoxycephalosporin-C synthase; DSAT, Desaturase; ECTO, Ectoine hydroxylase; FLAV, 2S-Flavones; GBBH, γ – butyrobetaine hydroxylase; GIAC, Gibberellic acid; HILY, Histone lysyl demethylase; HP4H, Hypoxia prolyl 4-hydroxylase; HYOS, Hyoscyamine; NUHY, Nucleotide/side hydroxylase; OGFD, Eukaryotic initiation factor 2α (eIF2α); PHYT, Phytanoyl-CoA; SULF, Sulfate cleaving; TFDA, 2,4-Diphenoxyacetic acid metabolizing; THYD, Thymidine dioxygenase; THYE, Thymine dioxygenase; XANT, Xanthine hydroxylase.

The taxonomic distribution of the sequences of DB2OG (Figure 2A), appropriately mirrors the assortment of biochemical reactions that typifies this superfamily. An interesting observation is the predicted cellular location of some of these sequences. While, the cytosol is the preferred location across all taxa, a considerable fraction of fungal proteins appear to be associated with the nucleus and the cytoskeleton when compared to animal and plant sequences. In comparison, animal 2OG-dependent enzymes seem to localize to the endosome (E.R. and Golgi apparatus), mitochondria, the plasma membrane, and the extracellular space. This perhaps reflects the need for glycosylation to achieve optimal activity with subsequent secretion (Figure 2B; Table T3). Analysis of the sequences specific to plants shows expected patterns. The vast majority are enzymes that participate in flavonoid metabolic pathways, gibberellic acid (GA) catabolism, 1-amino-1-cyclopropane carboxylate oxidase (ACC-oxidase; the terminal enzyme in ethylene synthesis), prolyl hydroxylases, alkaloid biosynthetic enzymes, and senescence related proteins, like the hormone regulator jasmonic acid (Figures 2C,D; Table T4). A large group of protein sequences which are either un- or minimally- annotated, or present nominally (PROB, *N* = 727), are grouped together, with probable roles in metal chelation, defense, and detoxification. Some of these proteins are predicted to complex iron directly, notably, the phytosiderophores (Nakanishi et al., 2000).

#### Distribution of catalytic domains in plant 2OG sequences

**Figure 3:**
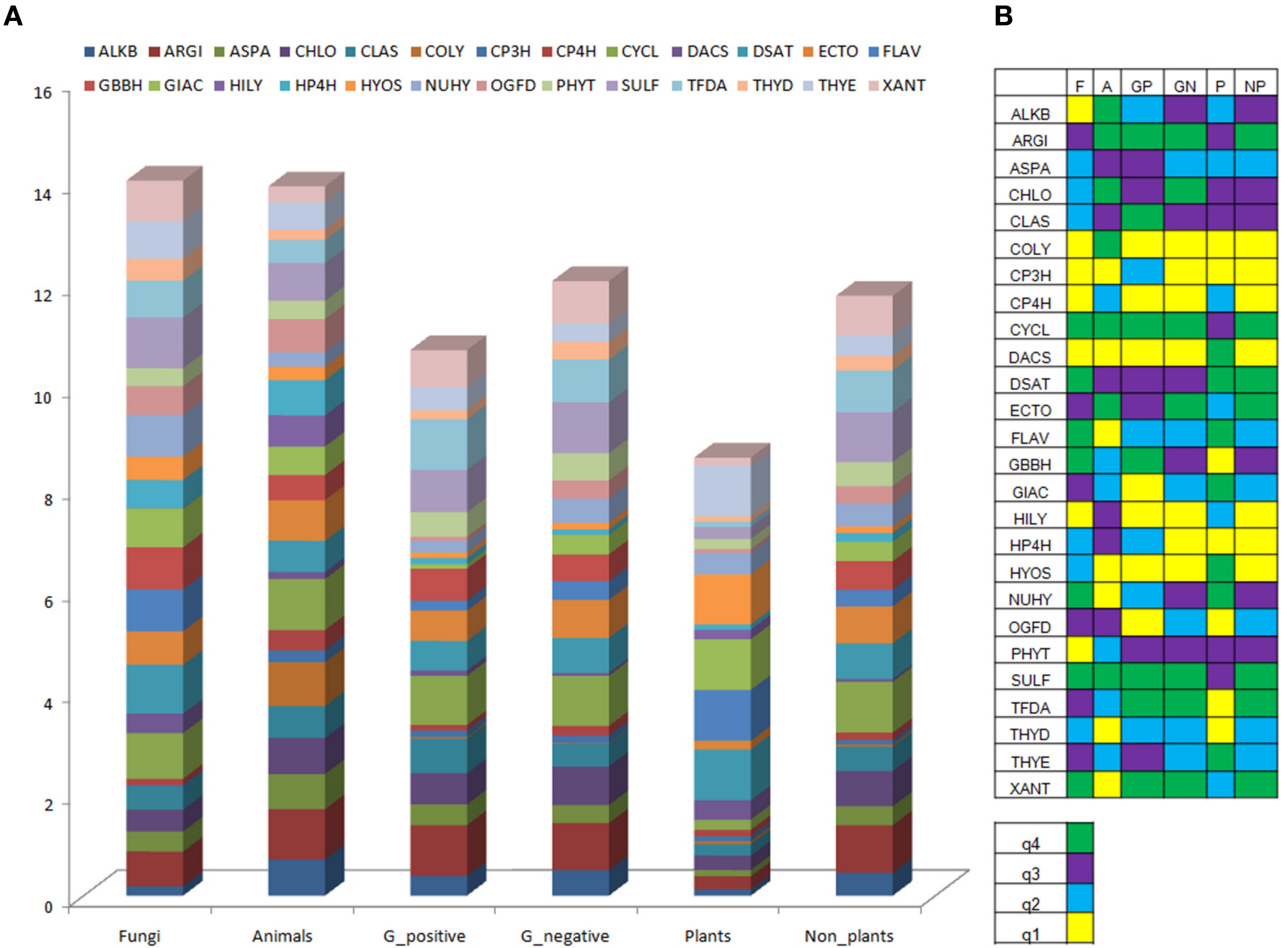
**Domain analysis of sequences of DB2OG. (A)** Taxonomic spread of catalytic domains in sequences, **(B)** Quartile representation of domains. Abbreviations: F, fungi; A, animals; GP, gram positive; GN, gram negative; P, plants and algae; NP, non-plants. Nomenclature for enzyme families, see abbreviations in Figure 2.

A comparative analysis of plant 2-oxoglutarate domains with sequences of other taxa (*N* = 1624; Figure 3, Tables T1, T2; S2A, S2B), conveys important information on the nature of enzymes that have specialized functions. The domains DACS (Deacetoxycephalosporin-S synthase/expandase), GIAC (Gibberellic acid metabolizing), HYOS/ALKO (Hyoscyamine transforming), NUHY (nucleotide/side hydroxylases), and THYE (Thymine dioxygenase) are exclusively high scoring (q4), in comparison to other organisms. Coupled mono- (DSAT; desaturase) or poly- (FLAV; 2S-flavones) hydrogen- abstracting, i.e., desaturases, have (q4) scores, but are shared with fungal spp. The domains SULF (sulfate cleaving), CHLO (chlorinating), and CYCL (cyclization) are uniformly high (q3, q4), in all sequences examined. In contrast, the low scoring plant GBBH (γ-butyrobetaine), COLY (collagen lysyl-dioxygenases), and TFDA (enzymes catalyzing the degradation of phenoxyalkanoic acid herbicides) (q1), and XANT domains (Xanthine hydroxylase; q2) have high valued equivalents (q4) in fungal, animal, and bacterial sequences. Homogenous low scoring CP3H (collagen prolyl 3-hydroxylase) and THYD (Thymidine dioxygenases) (q1, q2) were also part of this profile. These results suggest that while collagen lysyl-hydroxylases (COLY) predominate, expectedly in animal tissues, gibberellic acids (GIAC), alkaloids (HYOS), deacetyl/deacetoxycephalopsorin-C synthases/expandase domains (DACS) populate plant sequences. The susceptibility of plant DNA to undergo modifications is reflected by the presence of the NUHY and related THYE domains.

The combined domain frequency (α_*i*_; Table T2C) for a particular enzyme is the product of its occurrence within the plant sub-database and DB2OG, and is used in this work as a plausible measure of the catalytic domain spread within plant 2OG-dependent sequences.

### Statistical representation of 2OG-dependent sequences

#### Generalized linear model

A predefined objective function (***F***) is approximated by a linear combination of variables, a value which is determined by the number of enzymes hypothesized (Equation 1). Several case scenarios are listed wherein enzymes may be part of the same or distinct metabolic pathways, catalyze common or related substrates, or recognize specific molecular stereochemistry with the production of structural isomers (Table 1).

(1) $$F=\sum_{i\;=\;1}^{N\;=\;n}C_{i}X_{i}+E_{i}$$

(2) $$\begin{array}{l} \;C_{i}=-\log\left(\alpha_{i}P_{i}\left(\prod_{j\;=\;1}^{T\;=\;t}Km_{j}\right)\right)\\ \;\alpha_{i}:=\text{combined domain frequency}\\ \;P_{i}:=\text{probability of reaction occurring}\\ Km_{j}:=\text{average substrate affinity}\\ \;E_{i}:=\text{error term}\\ \;X_{i}:=\text{distinct generic metabolite}\\ \;n\;=\text{number of enzymes considered together}\\ \;t\;=\text{number of }Km\text{ values} \end{array}$$

**Table 1:** **Alpha-ketoglutarate dependent enzymes as mediators of synergistic function**.

	**Enzyme**	**Km (mM)**					
		**α^‡^**	**Iron (II)**	**2-oxoglutarate**	**Dioxygen**	**Substrates**	**References**
A	Prolyl 4-hydroxylase (HP4H)	0.11	0.0031	0.03	0.186	0.053	De Jong and Kemp, 1984; Hirsila et al., 2003; Kukkola et al., 2003; Myllyharju, 2003, 2008
B	Prolyl 4-hydroxylase (CP4H)	0.12	0.0031	0.021	0.04	0.311	Kivirikko and Myllyla, 1982; Kivirikko et al., 1989; Helaakoski et al., 1995; Lamberg et al., 1995; Annunen et al., 1997, 1998, 1999; Hieta et al., 2003; Kukkola et al., 2003; Myllyharju, 2003, 2008; Kersteen et al., 2004
C	Prolyl 3-hydroxylase (cis)	0.10		0.08		0.3975	Mori et al., 1997; Shibasaki et al., 1999, 2000
D	Prolyl 3-hydroxylase (trans)	0.10	0.0005	0.08	0.03	0.165	Tryggvason et al., 1979; Tiainen et al., 2008
E	Asparagine hydroxylase	0.17	0.0005	0.019	0.09	0.01	Hewitson et al., 2002; Koivunen et al., 2004; Flagg et al., 2012
^*^Subcase 1	A + E		***F*** = (3.0266)***X*1** + (4.3943)***X*2** + 0.9469				X1:= 4-hydroxyproline
							X2:= hydroxyasparagine
^*^Subcase 2	(A + B) + E		***F*** = (7.54375)***X*1** + (4.3943)***X*2** + 0.58821				X1:= hydroxyproline
							X2:= hydroxyasparagine
^*^Subcase 3	(A + B + C + D) + E		***F*** = (1.6997)***X*1** + (4.3943)***X*2** + 0.6779				X1:= hydroxyproline
							X2:= hydroxyasparagine

* *Coefficient details (S3): Subcase 1: X1:: se = 0.4515, p = 1.3E-09, df = 99; Subcase 2: X1:: se = 0.09281, p < 2.00E-16, df = 99; Subcase 3: X1:: se = 0.1825, p = 3.7E-15, df = 99; Subcases 1, 2, 3: X2:: se = 0.4954, p = 3.41E-14, df = 99*.

‡ *See Table T2C in Data sheet 2*.

#### Algorithm to compute coefficients

Step 1: Formulate the master equation.
Step 2: Take each term of *F*, i.e., *C*_*i*_ separately and use Equation (3).
  (3) $$\begin{array}{l} (\beta_{k})(C_{i})=\delta_{k}\\ \;C=\text{result from equations 1 and 2}\\ \;\beta ,\delta :=\text{random numbers (0, 1)}\\ \text{ k }:=\text{observation} \end{array}$$
Step 3: Iterate for 100 observations.
Step 4: Introduce the GLM formula and use the “quasi” family of distributions to compute and analyze the coefficients, significance, and the standard error.
Step 5: Sum the errors.

The formulation (Equations 1 and 2) ensures that the partitioning probability, domain frequency, and Km values to determine values of the *F*. The following scenario illustrates the significance and utility of this combinatorial approach.

**Case:** Role of prolyl 4-hydroxylase and asparagine hydroxylase in plant biology. Both these enzymes, acting together are known to mediate the hypoxia-response in mammalian tissues. Here, the model may be a minimalistic or conversely, represent all functioning enzymes in great detail. These variants are referred to as subcases and lead to the computation of different coefficients (Table 1).

#### Conformity to the indubitable

There are multiple ways to represent/model this biochemical federalism. Regression models (linear and non-linear) mandate the presence of a large corpus of raw data which could then be fitted to a suitable equation. The fit is assessed by examining the values of the coefficient of determination (*R*^2^). Here, model quality improves as *R*^2^ → 1. Although computationally intensive, polynomial equations (*f*(*x*) = *P*) of high degrees (*n* > 5) perform considerably better. Artificial neural networks (ANNs), which may consist of several layers of hidden nodes and a weight modifying function, need to be initially trained on existent datasets. The statistical model along with the computational algorithm outlined in this work is robust and theoretically implementable, compromising perhaps partially on the specificity for a particular system. The information used for the final calculations subsumes the frequency of catalytic domain occurrence (structural), numerical probability of a reaction, and Michaelis-Menten (Km/Ka) constants for several key substrates and cofactors (reaction chemistry). This basic formulation is followed by using the monte carlo method to predict a possible set of inputs (S4), which in turn is used by the GLM, as inputs to a family of distributions (poissons, gaussian, gamma, inverse gaussian, binomial) for comparative analysis. The data suggest the computed grand mean might serve as accurate estimators of the final coefficients (Table 1). The error values for a particular subcase are an aggregate of the standard errors of each coefficient predictor. Equation (1), computes a modified probability function that when used could be approximated by well-known probability density functions (PDFs). The subsequent parameterization could then used to glean salient features of the underlying system. Consider the subcases −1, −2, and −3 (Table 1), the enzymatic data suggests a progressive coarse graining (specificity → 0). In these cases studies a normal distribution provides the best fit. The selection of the random number from a given set of values (Gaussian/uniform) clearly highlighting the rationale of this approach. The system under study may then be mapped and investigated numerically.

### 2OG-dependent dioxygenase activity in regulating systems-level function in plants

**Figure 4:**
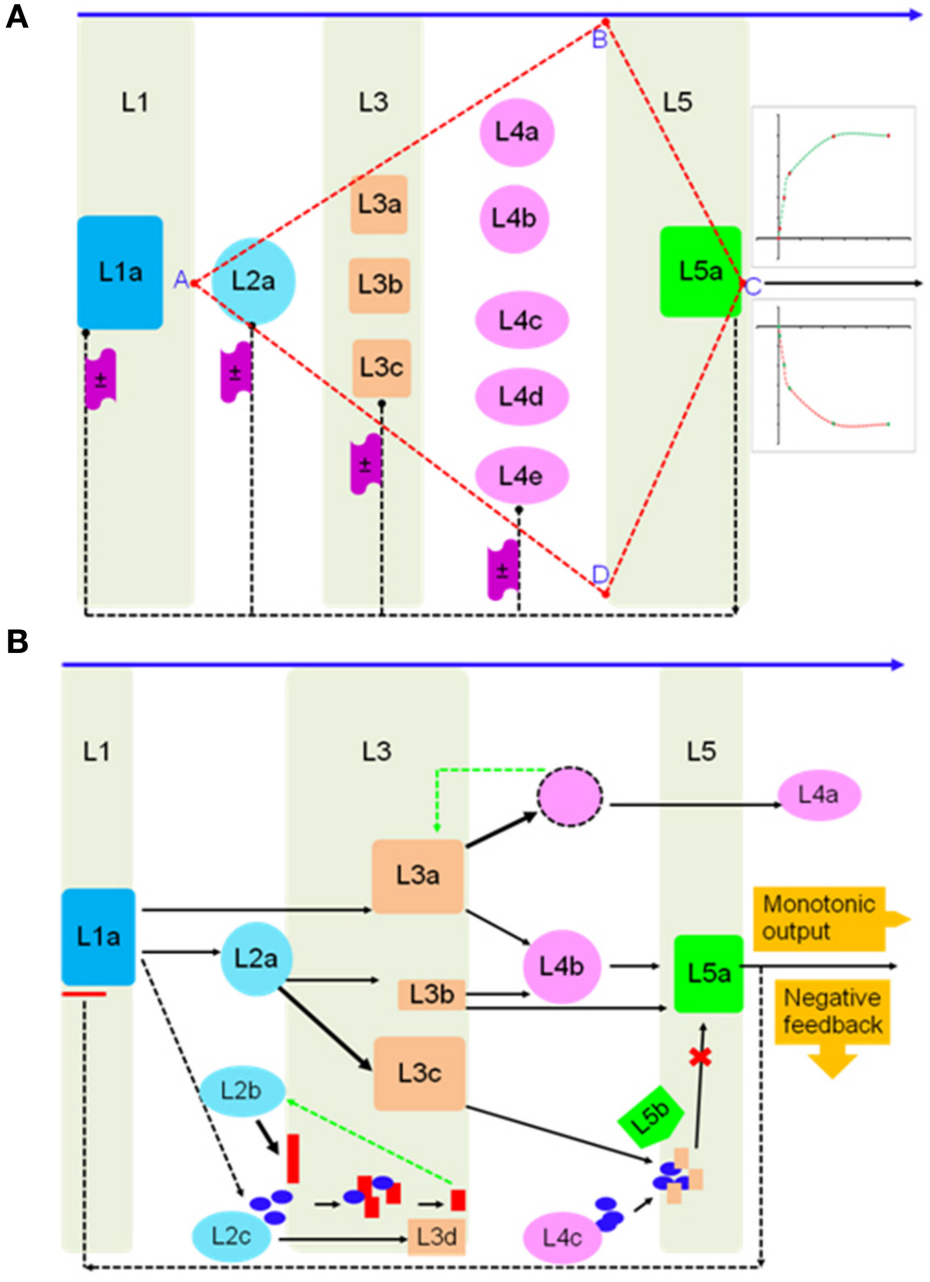
**System scalable architecture of a biochemical network in plant cells**. **(A)** Hypothetical multi-layer model of initiation, consolidation, and termination phases of a stimulus-motivated response. The arrangement is tuned to exhibit an amplification or fast-dampening of the resultant output, and **(B)** molecular models of network behavior, *viz*., feedback, competition, and bias, are implemented in a representation of weighted bi-directional information flow triggered by a declining essential nutrient(s).

Deploying a top-down strategy, a generalized system response can be fractionated into an interconnected sequence of distinct steps (*S* = ∫ *dx*; Figure 4A), arranged as part of a multilayered complex (L1–L5; Figure 4A). Here, the expression ***F*** (Equation 1) might be a thought of as a solution to the system-model (*S* ≅ *F* + *c*_0_). If, the constraint of a cascade were imposed (*AB* ∪ *AD*), downstream processes, which are disproportionate to the initiating stimuli can be embedded. Here, a cascade is defined as being scalable (λ), with factors for amplification or damping (*S* = ±λ ⨛ *dx*). Fine-steplets can be programmed by incorporating positive- (*L*2 ⇋ *L*3 ⇋ *L*4; Figure 4A) and negative-feedback mechanisms (*L*2*b* + *L*2*c* ⇋ *L*3*d*; *L*3*a* ⇋ *L*4*a*; Figure 4B). These would allow an element of robustness into the system making it less sensitive and more threshold dependent. Horizontal interconnectivity between individual steps may vary from competition for a single resource (shared metabolic intermediates; *L*2*a* → *L*3*b* ∨ *L*2*a* → *L*3*c*), to shared storage allocation nodes (nutritional reservoir; *L*3*b* → *L*4*b* ∧ *L*3*c* → *L*4*b*) (Figure 4B). Reversibility (sub-threshold), can be a function of nutrient deprivation/consumption or including negation equivalents of key parameters, while, plasticity or an irreversible change may be molded into the system by switching to amplification/termination mode.

The biological role of individual members of this superfamily of enzymes is unequivocal. However, given its taxonomic spread, conserved requirement of co-factors and—substrates, substrate and reaction chemistry heterogeneity, the systems level relevance of these enzymes remains speculative. In mammals, these enzymes might work to coordinate a transcriptional response to hypoxia, fatty-acid metabolism, and structural integrity, while in bacteria and fungi, antibiotic synthesis and pesticide breakdown may be the dominant roles. In plants the hormonal regulation of growth and development involving ethylene and gibberellins are well-characterized, as is the maintenance of rigid cell wall and transport of critical nutrients.

#### Monotonicity and substrate heterogeneity as pre-requisites for systems play

The archetypal melange of 2OG-dependent substrate turnover is crucial to their ascribed role as a systems player. Here, the distribution of catalytic domains in DB2OG has been utilized as an index of this variability. Other discrete measures take into account active site geometry and sequence homology as possible indices (Hausinger, 2004; Clifton et al., 2006; Kundu, 2012). The definition and prediction of domains used in this work conjoins structure-sequence data with the theoretically rigorous HMMs (Kundu, 2012), and is therefore a suitable measure of substrate and reaction heterogeneity. Manifestations of this could be a facultative requirement for 2OG (ACCO, EC 1.14.17.4; Rocklin et al., 2004; Zhang et al., 2004; **Figure 6A**), or an incomplete facial triad (SyrB2; Blasiak et al., 2006). However, 2OG-dependent dioxygenases are inherently bipolar. While at one extreme members possess a common jelly-roll fold, arranged as major (*N* = 7–10) and minor (*N* = 3–5) antiparallel β-strands, at the other is the breadth of substrate modifying action, secondary to an equally divergent reaction chemistry. This unifying structural signature suffices, in so far as the dependence on 2OG as an adjunct donor and requirement for Fe (II) is concerned, permitting the regulatory arm of this superfamily to be conserved, a factor that is essential to executing a systems-level response.

#### Sensing and triggering a response

**Figure 5:**
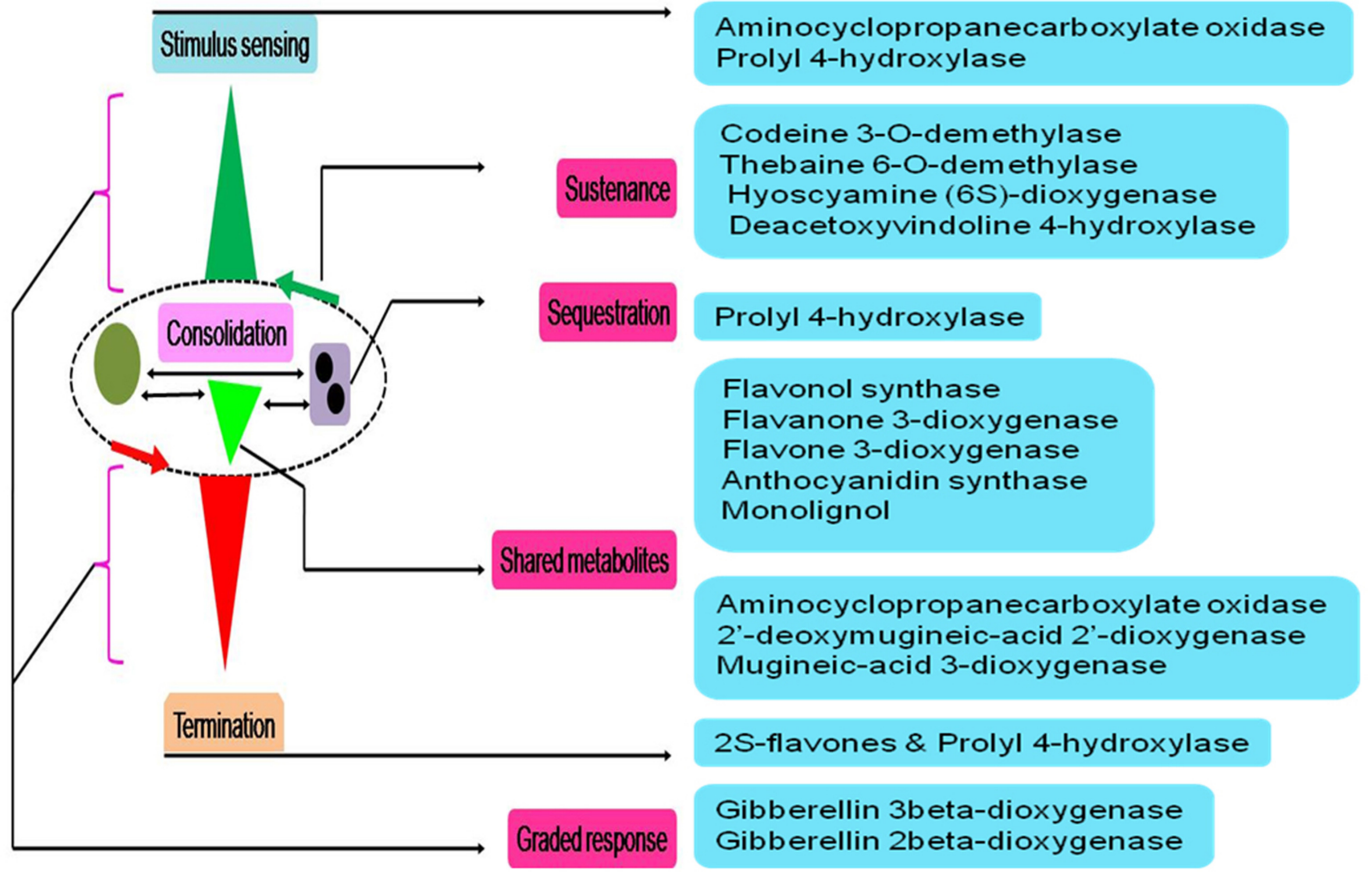
**Physiological model of 2-oxoglutarate dependent catalysis in influencing plant cell function**. These enzymes have well established roles as catalysts and possess an asymmetric cellular distribution. Any model that spans an activity profile requires multiple interfacing points. These levels could be active site-, reaction-, threshold-, or compartment-specific. Suitable examples from this superfamily, able to work in a concerted manner to accomplish a systemic role are highlighted.

A pan-systemic role for AKG-dependent dioxygenases may only be envisaged if members populate, and thereby, influence the cascade at all possible levels (Figures 4, 5). This would, in turn only be feasible if, supplemental to the variations discussed, *vide supra*, there were examples of graded responders to a particular stimulus or substrate. The enzymes prolyl 4-hydroxylase (P4HY; Figures 5, **7B**) and ACCO might serve to facilitate these effects as sensors and terminators of a particular response. An analysis of kinetic data for oxygen in P4Hs (EC 1.14.11.2) suggests that at Km values greater than 0.065 mM, the enzyme may function as a index for declining cytosolic oxygen (De Jong and Kemp, 1984; Hirsila et al., 2003; Myllyharju, 2008), while the higher affinity forms, i.e., <0.065 mM function as efficient catalysts (Hutton et al., 1967; Kivirikko and Myllyla, 1980; Tanaka et al., 1980; Chrispeels, 1984; Myllyharju, 2008). Another salient feature of P4Hs, at least in plants is the almost complete sequestration (≈85%; *N* = 22) of this enzyme in various organelles. Since, proline/hydroxyproline-rich glycoproteins (PRPs) are abundant (≈20%; Hijazi et al., 2014) in cell walls, this may impose a measure of sensitivity to 4-hydroxyproline levels. Any insult to P4H activity in the form of insufficient co-factors or transporters could weaken the cell wall, and initiate a downstream response.

The role of ACCO appears more complex. The presence of non-enzymatic product formation or facile electron transfers (FETs) ensures a steady production of ethylene gas (≅0.35 mol; Rocklin et al., 2004). The facultative role for 2OG and ascorbic acid in ACCO is puzzling given the conserved active site residues. An examination of the active site of the crystal structure (Zhang et al., 2004; PDB_ID: 1WA6), suggests that despite a relatively unconstrained and open structural core, narrow conformational pockets in the crystal structure of ACCO might favor binding of the more compact and abundant ACC (MM ~ 101.1 g mol^−1^), rather than AKG (MM ~ 146 g mol^−1^) to critical amino acids. ACCO also possesses a low Km (0.111–0.125 mM; Kosugi et al., 1997) for its cognate substrate (ACC). These factors may ensure a high active-site occupancy by ACC at all times with a resultant decreased need for 2OG.

#### Consolidating the response by sharing metabolic intermediates

To comprehend the importance of this stage a clear definition of what it entails needs to be outlined. Consolidation, here, describes a decrease in the probability of a reverse step. Viewed from thermodynamic principles, a decrease in entropy might be a suitable analogy. As in the case of supra-threshold stimuli, sub-threshold states, too may be considered bound by small intervals (δS). This would imply that progression to the next sub-interval dS(t) → dS(t + 1) is dependent on achieving this unidirectionality. Given, the multitude of overlapping reactions in a cellular mileau, this dynamic bias is a pre-requistite to any product formation or its consequences thereof. Absence of this reaction vector would render the system static and unresponsive, an undesirable outcome if stressor neutralization were the objective.

**Figure 6:**
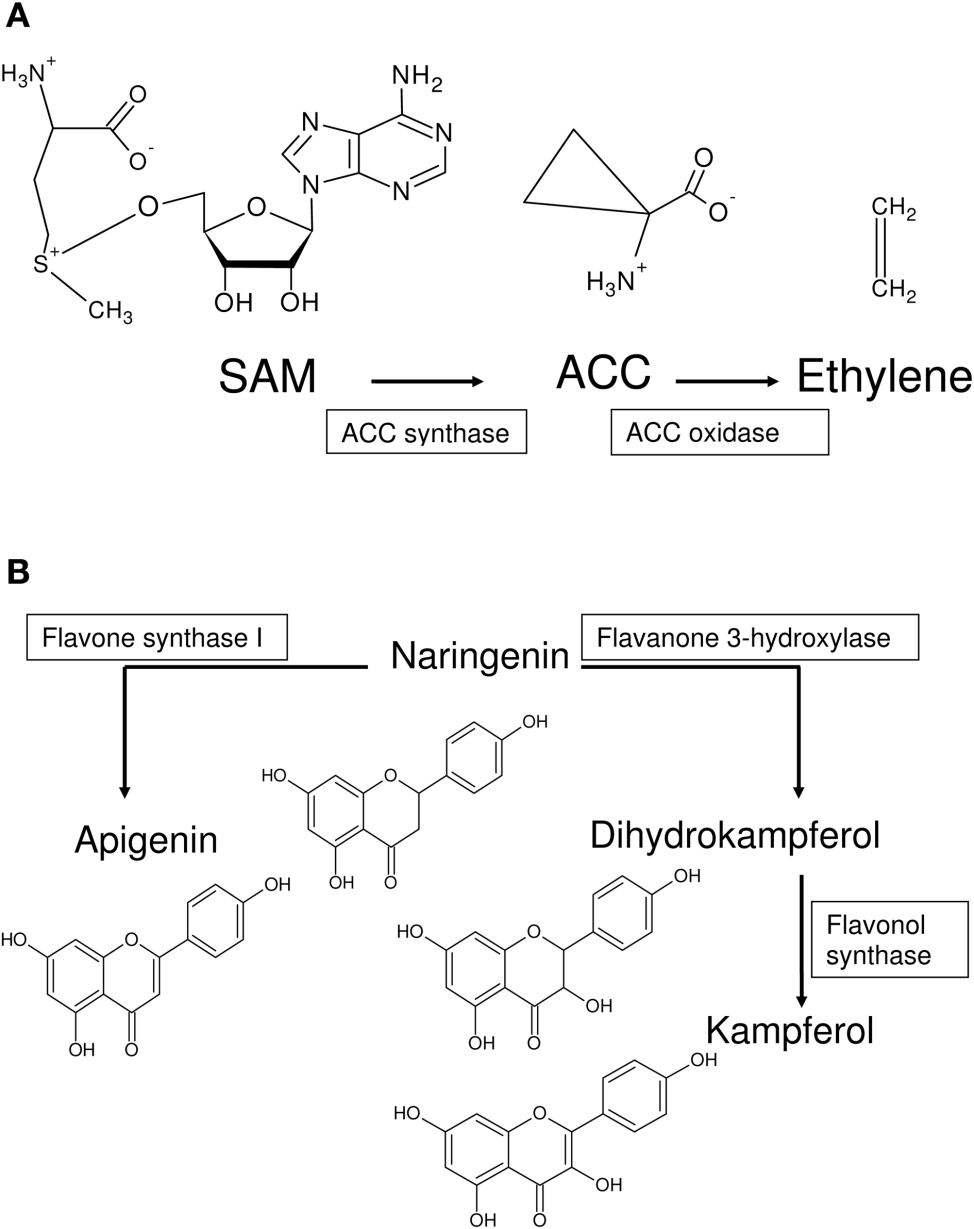
**Generation of metabolic bias in pathways utilizing 2OG-dependent enzymes**. **(A)** S-adenosylmethionine (SAM) is the substrate for ACCS, an EFE, and Nicotianamine (NA). In graminaceous plants, NA is converted to the mugineic acids. ACCO catalyzes the final step in the synthesis of ethylene, and maybe a critical player in the iron-deficiency sensing mechanism. **(B)** *p*-coumaroyl-CoA (not shown), is another metabolite at a branch point of two competing pathways. Naringenein, synthesized from CHS, is the first committed step in the synthesis and accumulation of flavonoids. The polyhydroxylated nature of these compounds and their metal chelation are important for their biological roles as antioxidants. The alternate metabolic route leads to the synthesis of lignins, which are used to strengthen the cell wall.

**Figure 7:**
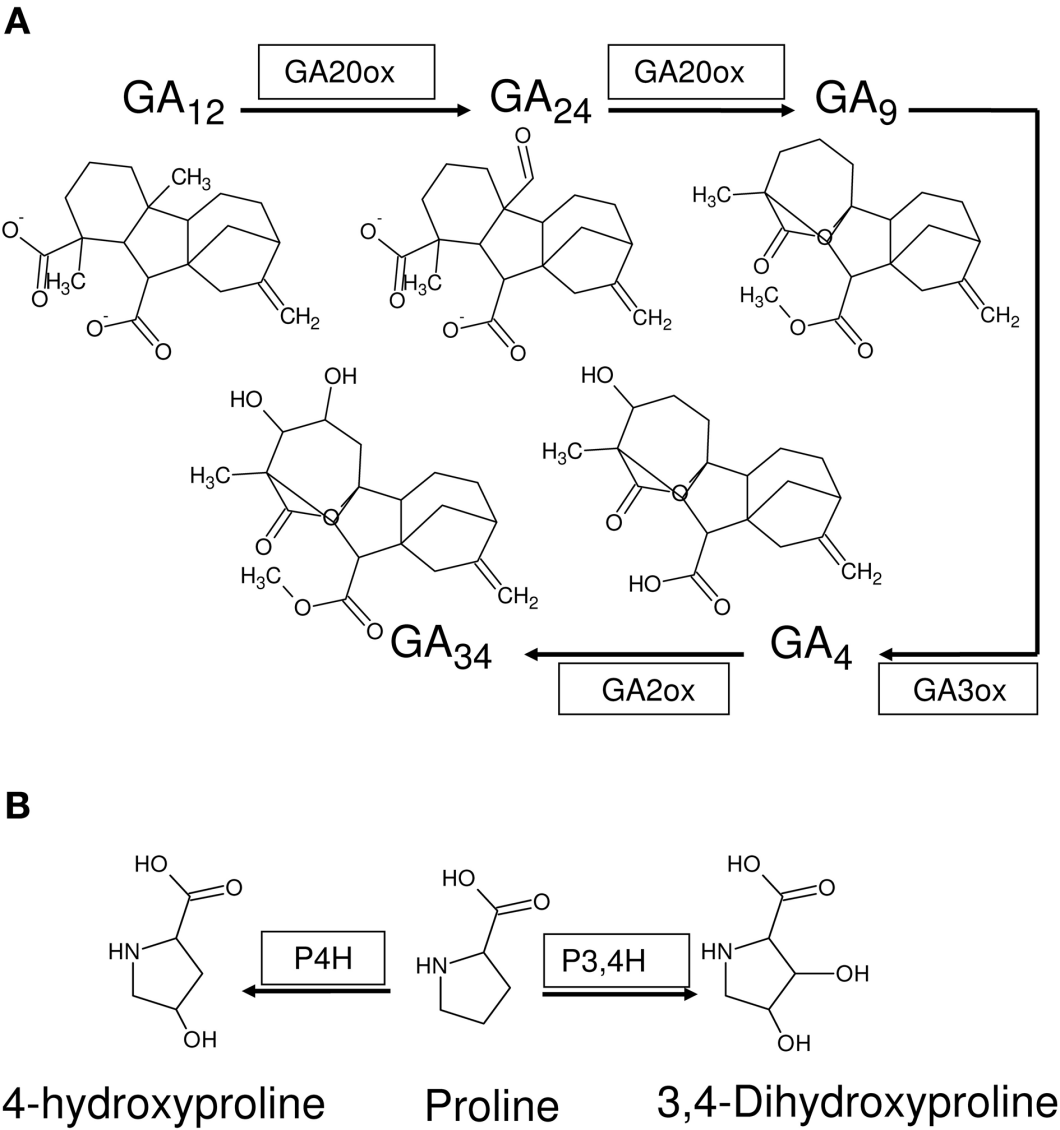
**2-oxoglutarate-dependent dioxygenases as stimulus reinforcers**. **(A)** Anabolic (GA20ox, GA3ox) and catabolic (GA2ox) alpha-ketoglutarate dependent enzymes function to prepare a cell mileau conducive to growth and development (increased formation and storage of alkaloids, expansins, glycosylases, and decreased formation of lignins) and a modulatory antioxidant process (formation of flavonoids). **(B)** Plant P4Hs catalyze the hydroxylation of proline-rich peptide segments of cell wall glycoproteins, stabilizing the macromolecule. Conversely, decreased activity might result in loosely networked unstable components, that are required for continued growth.

Four kinds of bias may be identified in 2OG-dependent plant dioxygenases (Figures 4, 5): (a) competing routes of reactions with shared substrate preferences. In the event of a flux toward a particular pathway, metabolites of the other decline reciprocally. The major biosynthetic channel of the flavonoids (Figure 6B), is the routing of *p*-coumaroyl-CoA through chalcone synthase (CHS, EC 2.3.1.74), in competition with the monolignol synthesizing hydroxycinnamoyl-CoA: shikimate/quinate hydroxycinnamoyl-CoA transferase (HCT, EC 2.3.1.133) (Burbulis et al., 1996; Hoffmann et al., 2004; Figure 6), (b) continued product utilization is an alternate strategy to favor certain reactions, thereby, introducing a dominant direction (*L*3*a* ⇋ *L*4*a*; Figure 4B). Activated L-methionine (S-adenosylmethionine, SAM) is the precursor for both ACC and nicotianamine (NA), which in the presence of Fe-deficiency, leads to the unhindered synthesis and efflux of the mugineic acids (MAs) which function as phytosiderophores (Nakanishi et al., 2000; Nozoye et al., 2011; Figure 6), (c) co-factor driven altered enzymatic activity of gibberellic acid (GA) metabolizing enzymes (GA −20, −3, −2 oxidases; EC 1.14.11.x, x = −, 15, 13) (Figure 7A). The kinetics (GA3O, Km_Fe_ ≅ 0.2 mM; GA2O, Km_Fe_ ≅ 1.0 mM) (Smith and MacMillan, 1984; Kwak et al., 1988), suggest that if cytosolic iron levels drop to levels below 1mM, the activity of the catabolic GA2ox approximates a null value. This balance between the highly active GA −1, −3, −4, −5, and −7 and the inactive GAs −8, −34, −97, −110 (Hedden and Phillips, 2000a,b) is critical to seed dormancy, root and shoot development, flowering, and generalized cell elongation. GAs serve as master regulators of other enzyme systems as well, and (d) initiate a cascade of reactions utilizing high affinity AKG-dependent enzymes for small molecule modifiers. Activated molecular dioxygen is a key component of these enzymes and could diffuse out if catalysis was compromised. The active site geometry of ACCO (Km_Fe_ ≅ 0.059 mM; Nagahama et al., 1991) not just permits FETs, but may mitigate the effects of co-substrate withdrawal as well. The free radicals generated subsequently by O_2_ (ROS and RNS) amplify the response manifold. The rapid generation and involvement of substrate radicals (lipid peroxidation of polyenes), could translate into an equally swift consumption of potential substrates, which indirectly, could introduce the requisite bias.

The aforementioned discussion results in a systemic response that is committed and increasingly ordered. Preservation of the division between system-elasticity and -plasticity is co-terminus with evolution of complex systems and translates into a reversibility factor whose mathematical limit approximates zero. This lower bound can be realized biochemically by sets of reactions that negate each other or simply exhaust an important resource.

#### Sustaining/terminating the response for/after a finite duration

The reversibility of any path is integral to regulating a response proportionate to the stimulus. AKG-dependent proteins working together, and with other exponents of cell function can facilitate the near-cessation of a response (Figures 4, 5). In most cases this necessitates sophisticated feedback mechanisms embedded in the implementing nodes.

**Figure 8:**
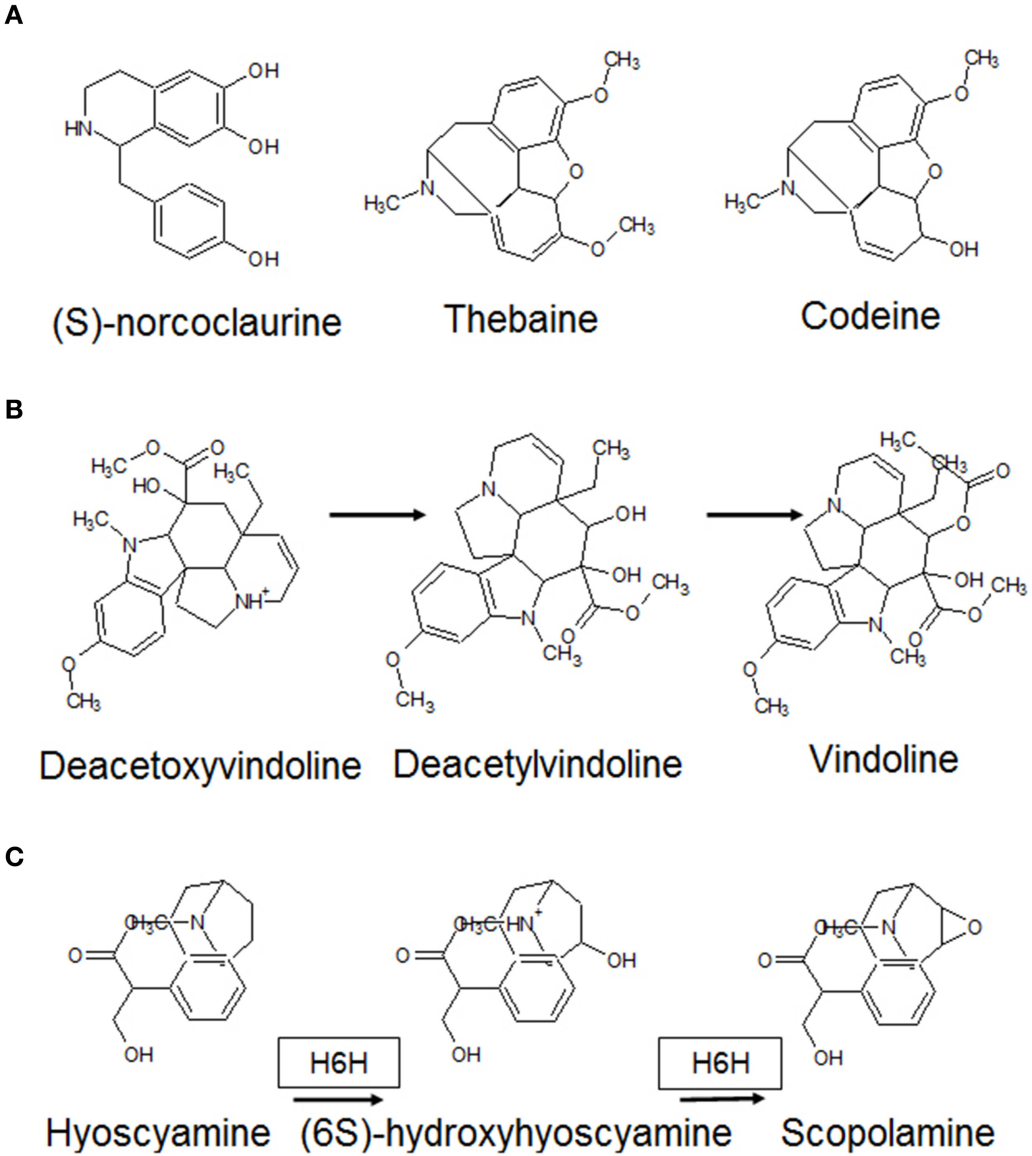
**Alkaloid classes that require 2-oxoglutarate as a co-substrate**. These N-heterocyclic molecules are often synthesized in preparation for growth of new tissue and serve as an on-demand nitrogen source accessible at all times. As the tissues grow and differentiate, alkaloid reserves have been shown to progressively deplete. **(A)** (S)-norcoclaurine is the precursor of the isoquinoline classs of alkaloids (codeine, thebaine). These require demethylases, which interconvert them. **(B)** The vinca alkaloids (precursor: deacetoxyvindoline), are inhibitors of microtubule transport and terminally synthesized compounds (vincristine, vinblastine) are used in various chemotherapy regimens. **(C)** The tropanes are best represented by hyoscyamine and scopolamine both of which require activity of H6H (Hyoscyamine 6β-hydoxylase).

These may be intermittent (fluctuate) or persistent (uniform). A fluctuating system is dependent on the presence of a connection between the output and input. Here, the magnitude of difference between the observed and desired outputs is funneled back to the input signal, which may be modeled as a weighted variable corresponding to the factor(s) in question (ANNs, artificial neural networks) with backpropagation- (negative) modulation. The efflux of MAs with the resultant solubilization of Fe (III) chelates in the rhizosphere and transport-protein mediated divalent cation import would automatically constitute a self-limited closed loop by restoring cytosolic iron levels. A resilient framework (ANN with positive- or negative-feedforward moderation), however, would be dependent on *de novo* synthesis or conversely an exhaustible pool of compounds. These events might be expected to dominate proceedings at a later stage in the response. Major contributors within the plant 2OG-dependent dioxygenases conforming to this model are alkaloid biosynthesis, the free radical pathway, and prolyl 4-hydroxylases (Figure 5). Alkaloids are nitrogenous compounds produced by many organisms, and possess distinct pharmacological profiles. Analysis of DB2OG for probable alkaloid-synthesizing enzymes in plants suggests that the isoquinoline (thebaine, codeine), monoterpenoid indole (vinblastine, vincristine), and tropane (hyoscyamine, scopoalmine) families of compounds could be deployed as a stable, local, and perishable pool of nutrients. (Matsuda et al., 1991; Vazquez-Flota et al., 1997; Hagel and Facchini, 2010a,b) (Figure 8). As mentioned earlier, oxygen, post-activation is a potent generator of free radicals, capable of scaling-up a response (Muller et al., 2009). Unchecked, these may cause an irreversible and unrecoverable loss of function. Besides the inter-radical neutralization, the upregulated polyhydroxylated 2S-flavonols previously synthesized (*L*3c → *L*5*b*; Figure 4B), could reprise their roles as powerful antioxidants (Kumar and Pandey, 2013).

The single-most critical end-point is the integrity of the cell wall. Interestingly, the local concentration of the principal components of both the primary and secondary cell wall can be assumed to be minimally supra-threshold, if not, borderline. This paradoxical vulnerability to biochemical factors is in contrast to the structural stability exhibited in transporting water and nutrients to various parts of the plant. Whilst, the sequestration of the P4HY could potentially effect the PRPs in the primary form, the sub-optimal monolignol synthesizing pathway weakens the molecular framework of the secondary cell wall (Gallego-Giraldo et al., 2014). Endoperoxidation of the membranous unsaturated fatty acids, elevated transcription levels of expansion proteins (EXPs; Cho and Cosgrove, 2002) and hydrolytic enzymes (hydrolases/transglycosylases/mannoses; Carpita and Kanabus, 1988; Cui et al., 2005) complement these to dismantle the cell wall. Senescence triggered by ethylene too, is purported to be the result of low pH conditions and/or elevated expression of certain expansin proteins (Vreeburg et al., 2005), and its continued formation, in tandem with other factors imparts a predisposition to cell wall weakening.

## Current status and future directions

There is a vast body of literature highlighting individual enzymes of this superfamily, and is genomic, biochemical, and structural in character. This work underscores a novel treatment for 2-oxoglutarate-dependent dioxygenases, i.e., as a systems player and digresses, considerably from the single enzyme single function norm. From this analysis, there seems to clear indication that this superfamily possesses the necessary credentials to transcend their well-characterized roles as supercatalysts. The evolution of this collegial behavior, from single molecules to mediators of complex function, however remains unanswered. The redundancy exhibited, in terms of absolute numbers may contribute to the development of biological hysteresis, and thresholds, on one hand, as well as permit fine tuning of response pathways to neutralize/mitigate noxious stimuli. Whether, these bounds could be used as predictors for the emergence of a complex system needs to be investigated further.

It is generally accepted that the presence or absence of a few residues can markedly alter the catalytic profile of an enzyme, both, abrogating as well as accentuating activity. This minimalistic notion has numerous proponents and a detailed treatment of this nano-/femto-level stochasticity could offer insights into enzyme association/dissociation and the consequent kinetics. The creation of a carefully curated resource of enzymes with probable 2-oxoglutarate-dependency will aid workers in profiling new members of this remarkable superfamily.

## Author contributions

SK collated the data, carried out the computational analysis, formulated and refined the models, constructed DB2OG and the GUI, wrote the code, and the manuscript.

### Conflict of interest statement

The author declares that the research was conducted in the absence of any commercial or financial relationships that could be construed as a potential conflict of interest.

## Abbreviations

2OG: 2-oxoglutarate
AKG: α-ketoglutarate
DB2OG: database of 2-oxoglutarate-dependent dioxygenases
HMM: hidden Markov model
GLM: generalized linear model
GUI: graphical user interface
ROS: reactive oxygen species
RNS: reactive nitrogen species.
